# COVID-19 and Smoking: What Evidence Needs Our Attention?

**DOI:** 10.3389/fphys.2021.603850

**Published:** 2021-03-18

**Authors:** Jianghua Xie, Rui Zhong, Wei Wang, Ouying Chen, Yanhui Zou

**Affiliations:** ^1^School of Nursing, Hunan University of Chinese Medicine, Changsha, China; ^2^Hunan Cancer Hospital, The Affiliated Cancer Hospital of Xiangya School of Medicine, Central South University, Changsha, China

**Keywords:** COVID-19, smoking, tobacco, electronic cigarette, viral transmission

## Abstract

The current COVID-19 pandemic has caused severe morbidity and mortality worldwide. Although relevant studies show that the smoking rate of COVID-19 patients is relatively low, the current smoking status of people with COVID-19 cannot be accurately measured for reasons. Thus, it is difficult to assess the relationship between smoking and COVID-19. Smoking can increase the risk of severe COVID-19 symptoms and aggravate the condition of patients with COVID-19. Nicotine upregulates the expression of ACE2, which can also increase susceptibility to COVID-19, aggravatiing the disease. Although nicotine has certain anti-inflammatory effects, there is no evidence that it is related to COVID-19 treatment; therefore, smoking cannot be considered a preventative measure. Furthermore, smokers gathering and sharing tobacco may promote the spread of viruses. Despite the COVID-19 epidemic, the findings suggested that COVID-19 has not encouraged smokers to quit. Additionally, there is evidence that isolation at home has contributed to increased smoking behavior and increased quantities. Therefore, it is recommended that governments increase smoking cessation messaging as part of public health measures to contain the COVID-19 pandemic. This review analyzes the existing research on smoking’s impact on COVID-19 so that governments and medical institutions can develop evidence-based smoking-related prevention and control measures for COVID-19.

## Introduction

Coronavirus disease 2019 (COVID-19) is caused by an infection from a new type of coronavirus. The virus was named the severe acute respiratory syndrome coronavirus 2 (SARS-CoV-2) by the International Committee on Taxonomy of Viruses (ICTV), like SARS-CoV and MERS-CoV, and it belongs to the Betacoronavirus genus (β-COV) (Graham et al., 2013; Song et al., 2019). The genetic sequence of SARS-CoV-2 is 79.5% similar to SARS-CoV, and the receptor used by SARS-CoV-2 to enter the human body is the same as that used by SARS-CoV, namely, the angiotensin-converting enzyme 2 (ACE2) (Lu et al., 2020). However, the binding affinity of the spiked envelope of SARS-CoV-2 to human ACE2 is approximately 10–20 times that of SARS-CoV, with an extremely high rate of human-to-human infection and rate (Wrapp et al., 2020; Yu et al., 2020).

From December 2019, it took only a few months for the new coronavirus, which causes pneumonia, to spread from sporadic regional outbreaks to countries to worldwide. The number of patients affected, the seriousness of the disease, and the high mortality rate are unprecedented. As of February 21, 2021 (Beijing time), the latest report of the World Health Organization (WHO) showed that the number of confirmed cases of new COVID-19 worldwide was more than 111.69 million people (111,696,136), with an estimated 2.46 million deaths. The United States (with approximately 28.67 million cases) and India with (approximately 10.99 million cases) are reportedly the most severely affected countries. There is still no effective treatment for COVID-19.

At this stage, the clinical characteristics of patients with the new coronavirus are still being explored in-depth; moreover, epidemiological data show higher morbidity and mortality rates from COVID-19 among the older adults and those with lower immunity and prior illnesses (cancer, hypertension, diabetes, and especially respiratory diseases) (Du et al., 2020; Salako et al., 2020). Smoking is a major risk factor for common chronic diseases (Tonnesen et al., 2019), especially those closely related to the occurrence and development of respiratory diseases. During the COVID-19 epidemic, the role of cigarette smoking on COVID-19 has been a controversial issue. This article reviews the latest research and evidence on smoking and COVID-19 to be used as a reference by medical institutions and clinicians.

## Smoking Increases the Risk of Viral Respiratory Infections

COVID-19 is primarily transmitted through the respiratory tract (saliva), and smokers may be at increased risk of contracting the virus due to reduced lung function, impaired immune systems, cross-infection, and susceptible hygiene habits (Zhou et al., 2016; Ahmed et al., 2020). Cigarette smoking also increases the amount of forced vital capacity (FVC) and stimulates hyperproliferation of the bronchial mucosal glands, resulting in increased mucosal permeability, excessive mucus production and inhibitedclearance of mucosal cilia, reducing the airway purification function and harmful microorganisms screening in the upper respiratory system, leading to potential pulmonary inflammation.

In a prospective study that explored smoking and alcohol consumption’s suppression of host resistance to viral infections, 391 participants were exposed to five respiratory viruses (including coronavirus type, respiratory syncytial virus and three rhinovirus types rhinovirue). The results showed that smoking increased the risk of infection (OR = 2.23; 95% CI: 1.03–4.82), as well as the risk of developing clinical symptoms after infection (OR = 1.83; 95% CI: 1.00–3.36) (Cohen et al., 1993). Another cell experimental study showed that when infected by the virus, cigarette smoke extracts preconditioned RSV-infected cells to cause cell necrosis rather than apoptosis, resulting in increased inflammation and increased viral replication (Groskreutz et al., 2009). Thus, smoking can increase the risk of viral respiratory infections.

A systematic review analyzed the current evidence and quantified the risk of influenza infection between smokers and non-smokers. Nine studies with a total of 40,695 participants were included in this review, of which three were laboratory-confirmed case-control studies of influenza showing that current smokers were 5 times more likely to develop influenza than non-smokers (OR = 0.73; 95% CI: 0.73–0.99). In six studies reporting the occurrence of influenza-like illness, current smokers were 34% more likely to have influenza than non-smokers (OR = 1.34; 95% CI: 1.13–1.69) (Lawrence et al., 2019). Jaspers et al. (2010) reported that in the early stage of influenza virus infection, the antiviral defense ability of smokers’ nasal epithelial cells was inhibited, namely, the signal transduction of type I interferon (IFN) was inhibited, IFN-α and IRF7 (a key transcription factor controlling the expression of IFN-α) expression was reduced, which would increase smokers’ susceptibility to the influenza virus. Meanwhile, Noah et al. (2011) suggested that the inhibition of type 1 IFN signal transduction would facilitate the replication of the influenza virus in smokers and individuals exposed to tobacco smoke. Therefore, it would increase the number of influenza viruses.

## The Epidemiology of Smoking and COVID-19

The WHO stated that 1.4–18.5% of hospitalized COVID-19 adult patients were smokers (World Health Organization, 2020a). Some scholars believe the prevalence of COVID-19 in Chinese men was higher than among women because the smoking rate among Chinese men was much higher than women (Underner et al., 2020). Some studies do not support the above conclusions. Farsalinos et al. (2020) conducted a systematic analysis of 13 studies from China (including 5,960 patients) indicating the current prevalence of smoking among hospitalized patients with COVID-19 was 6.5% (95% CI: 4.9–8.2%) based on a pooled estimate; In the secondary analysis, the unknown data were adjusted (integrating former smokers into the group of current smokers), and the pooled estimate of smoking prevalence was 7.3% (95% CI: 5.7–8.9%), which is still far lower than the prevalence of smoking among Chinese residents (26.6%). Tsigaris and Teixeira da Silva (2020) conducted an ecological study of 38 European countries, and after strictly controlling for confounding factors, smoking prevalence was significantly negatively correlated with COVID-19 prevalence (*P* = 0.001). Furthermore, a meta-analysis of 233 studies showed (Simons et al., 2020), current smokers compared with never smokers were at reduced risk of testing positive for SARS-CoV-2 infection (RR = 0.74; 95% CI: 0.24–0.64); But former smokers compared with never smokers were at increased hospitalization risk (RR = 1.20; 95% CI: 0.06–0.37).

Some researchers suggested that studies on smoking and COVID-19 have similar limitations, namely, they cannot accurately determine people’s current smoking status (Emami et al., 2020; Harapan et al., 2020; Guan et al., 2020; Miyara et al., 2020; Petrilli et al., 2020). There were significant differences between these incomplete patient health histories and actual smoking behavior, leading to the underestimation of current smoking rates of COVID-19 patients, which also caused a certain deviation in the early evaluation of the COVID-19 infection rate and smoking status (Polubriaginof et al., 2018; Lippi et al., 2020; Patanavanich and Glantz, 2020). Exposure to SARS-CoV-2 was heterogeneous, with higher infection risk in different subgroups at different stages of the pandemic, and some research analyses are based on unadjusted ORs (calculated for age and other confounding factors) (Feldman and Anderson, 2013; Leung et al., 2020; Lippi and Henry, 2020; Liu et al., 2020; Zhong et al., 2021), even some peer-reviewed meta-analyses investigating the association between smoking and COVID-19 were based on unadjusted ORs (Lippi and Henry, 2020; Zhao et al., 2020; Zheng et al., 2020). Therefore, the reliability of these studies needs to be confirmed. Also, smokers were more likely to have symptoms similar to COVID-19, such as cough and sputum, making them more likely to accept SARS-CoV-2 testing, even if they may not be infected, this included with the object selection bias would increase the negative samples detection rate, creating a bias (Cole et al., 2010; de Lusignan et al., 2020). Thus, the single smoking rate of patients cannot be used to judge the relationship between smoking and COVID-19, scientists need to be cautious in assessing the impact of smoking on COVID-19.

## Smoking and COVID-19 Disease Prognosis

In April 2020, the WHO announced that smokers were more likely to develop serious illness from COVID-19 than non-smokers (World Health Organization, 2020b). More studies have shown that smoking is associated with the prognosis of COVID-19 infection, and current and former smoking is significantly associated with the risk of serious illness from COVID-19 (Grundy et al., 2020; Liu et al., 2020). During previous influenza outbreaks (MERS-CoV), smokers were twice as likely to be infected with influenza and had more severe symptoms as non-smokers, and had a higher mortality rate (Arcavi and Benowitz, 2004; Park et al., 2018). Vardavas and Nikitara (2020) reported that from the initial original study of COVID-19 in China, smokers were 1.4 times more likely to have severe COVID-19 symptoms than non-smokers (RR = 1.4; 95% CI: 0.98–2.00), and smokers were approximately 2.4 times more likely to be admitted to the intensive care unit (ICU). Smokers may require mechanical ventilation more often than non-smokers or they may be more likely to die. In a single-center study, Zhao et al. (2020) showed that on-going smoking increased the risk of developing severe COVID-19 by approximately 2-fold (OR = 1.98; 95% CI: 1.29–3.05), and COPD patients who developed severe COVID-19 symptoms had a >4-fold increased risk (OR = 4.38; 95% CI: 2.34–8.20). However, the analysis of the subgroup revealed (the largest sample size was excluded for a sensitivity analysis) that the effect of active smoking on COVID-19 severity was no longer significant. In COVID-19 patients with chronic diseases, a history of respiratory and cardiovascular diseases accelerated deterioration, and smoking was closely associated with the development of these diseases (Wang B. et al., 2020; Wang X. et al., 2020).

**FIGURE 1:**
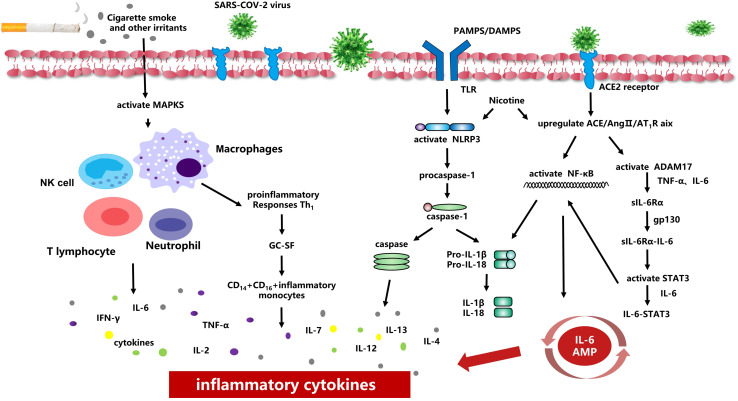
Smoking and inflammatory cytokines Smoking causes activation of MAPKs in the lung, increasing the number of neutrophil granulocytes, lymphocytes, macrophages and other cells, and inducing cells to release pro-inflammatory factors and chemokines. Smoking induces overexpression of ACE2 receptor and increases viral adhesion. When the virus is activated and released, NF-κB-mediated signaling pathways are activated, and thus up-regulated the transcription of inflammasomal-related components. Smoking can upregulate ACE/Ang II/AT1 raxis, activate NF-κB, integral protein and ADAM17, induce TNF-α and IL-6 to the soluble form sIL-6Rα, and finally activate IL-6 AMP to promote the release of inflammatory cytokines.

Most of these studies confirmed that cigarette smoking can aggravate COVID-19 symptoms in patients, which may be caused by a variety of chronic diseases related to smoking, especially because smoking can damage lung function and the immune system, causing a reduced ability to fight COVID-19. Cigarette smoking also causes excessive airway secretions, impaired drainage during expectoration, and impaired ventilation function, all of which increase susceptibility to acute respiratory tract dysfunction due to sudden phlegm asphyxiation. Furthermore, cigarette smoke can activate the autophagy-dependent mechanism mediated by the deacetylase HDAC6, while causes the cilia proteins to be delivered to the lysosome for degradation or recycling, shortening the airway cilia (Lam et al., 2013). The excessive activation of autophagy may eventually lead to programmed epithelial cell death, contributing to impaired mucociliary clearance (Cloonan et al., 2014). It can also reduce the A-kinase anchoring protein expression (AKAP)-9 *in vitro* to cause e-cadherin-mediated barrier function destruction of airway epithelial cells, leading to increased infection risk by viruses and bacteria that exacerbate COPD (Oldenburger et al., 2014). At the same time, cigarette smoking activates MAPKs in the lung, increasing the number of neutrophil granulocytes, lymphocytes, macrophages, and other cells, and inducing cells to release pro-inflammatory factors and chemokines in Figure 1 (Barnes, 2009; Chung, 2011). This results in acute lung inflammation, such as neutrophil infiltration, the mRNA expression of TNF-α and MIP-2, proteinase expression (MMP-12 mRNA), and oxidative DNA damage, causing respiratory barrier dysfunction and epithelial cell death. It also promotes the development of chronic inflammation in COPD and other related diseases (Marumo et al., 2014; Aghapour et al., 2018).

## Nicotine Affects ACE2 Expression, Increases Susceptibility to COVID-19, and Causes Aggravation of the Disease

The angiotensin-converting-enzyme II (ACE2) receptor is the confirmed entry point of the SARS-CoV-2 virus into host cells because the S1 domain of the SARS-CoV-2 virus membrane spike protein has a high affinity with the ACE2 receptor on lung epithelial cells (Kaur et al., 2020). ACE2 expression plays a key role in susceptibility to COVID-19 and is involved in innate and adaptive immune responses, affecting the immune regulation of B cells and cytokine secretion (e.g., IL-1, IL-10, IL-6.). Its high levels of expression may increase viral activity and promote viral replication, transcription, and release (Li G. et al., 2020).

In the process of virus replication and transcription, the combination of SARS-CoV-2 and ACE2 may progressively downregulate the expression of ACE2 protein and reduce the residual ACE2 activity (Dijkman et al., 2012), causing the renin-angiotensin system (RAS) to be unregulated, and then changing the ACE/ACE2 Balance and producing higher ACE and/or lower ACE2, while angiotensin-converting enzyme (ACE)/angiotensin (ANG) II/Ang II type I receptor (AT_1_R) axis could causing vasoconstriction, pro-inflammatory and pro-oxidative effects. It could eventually evolve into acute heart failure, ARDS, and renal acute failure (Garami, 2020; Henry et al., 2020).

**FIGURE 2:**
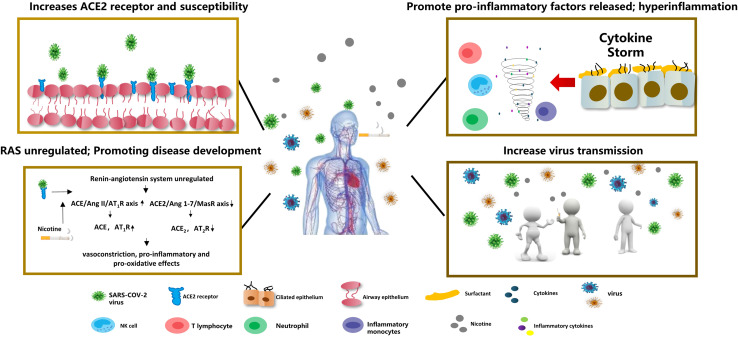
COVID-19 and smoking Nicotine induces overexpression of the ACE2 receptor in lung epithelial cells, increasing SARS-COV-2 virus susceptibility. Nicotine can also deregulate the renin-angiotensin system (RAS), leading to vasoconstriction, proinflammatory, and pro-oxidative reactions. Meanwhile, nicotine can increase the release of pro-inflammatory factors and aggravate the inflammatory response. Smokers’ social behaviors for gathering to smoke can increase the transmission of COVID-19.

Studies have confirmed that nicotine can affect the homeostasis of the renin-angiotensin system. It can upregulate the ACE/Ang II/AT_1_R axis and increase renin expression or activity, ACE and AT_1_R, while the ACE2/Ang 1-7/Mas receptor axis can downregulate the expression or activity of ACE2/AT_2_R in Figure 2 (Russo et al., 2020). The imbalance of the RAS system caused by nicotine may promote and aggravate the occurrence and development of cardiovascular, cerebrovascular, and pulmonary diseases.

ACE2 expression is regulated by nicotinic acetylcholine receptors (nAChRs). The nAChRs receptors are widely distributed throughout the central nervous system and activate acetylcholine neurotransmitter signaling pathways, while nAChRs can be easily activated by nicotine (Dani and De Biasi, 2001). When people smoke, nicotine induces overexpression of the ACE2 receptor in pulmonary epithelial cells (e.g., bronchial epithelial cells, type II alveolar epithelial cells.) through nAChRα7 (Biron et al., 1969; Wang Q. et al., 2020), and studies have confirmed that smokers (including e-cigarettes) have higher serum levels of ACE-2 in Figure 2 (Brake et al., 2020). In healthy human volunteers, serum ACE activity increased significantly immediately after smoking and returned to control levels 20 min after smoking cessation (Kitamura, 1987).

Farsalinos et al. (2020) believe that upregulation of ACE2 expression may be induced as a defense mechanism against the effects of angiotensin II. In animal experiments, ACE2 protected mice from severe acute lung damage (Imai et al., 2005), while tobacco smoke exposure increased lung injury in ACE2 knockout mice (compared with wild-type mice) (Huang et al., 2016), further supporting the upregulation of ACE2 as a beneficial defense mechanism. Smith and Sheltzer (2020) reported that smoking causes a dose-dependent upregulation of ACE2 expression; that is, long-term smoking increases ACE2 expression, while smoking cessation decreases ACE2 levels in the lungs.

Even if upregulation of ACE2 in the initial stage of tobacco exposure helps prevent acute lung injury, there is no doubt that upregulation of ACE2 expression can increase COVID-19 susceptibility, and long-term tobacco exposure can cause an imbalance of the body’s RAS system, leading to a series of changes. Thus, it progresses the COVID-19 disease.

## Nicotine, IL-6 and Other Pro-Inflammatory Factors

One of the main causes of death from COVID-19 is acute respiratory distress syndrome (ARDS), and the main mechanism of ARDS is the “cytokine storm,” which is caused by immune effector cells releasing large amounts of pro-inflammatory cytokines (e.g., TNF-α, IFN-γ, IL-1β, IL-6, IL-12.) and chemokines (e.g., CCL2, CCL3, CCL5) (Li X. et al., 2020). Cytokine storms can activate the immune system’s attack on the body, triggering a deadly and uncontrollable systemic inflammatory response that can cause acute respiratory distress and multi-organ failure (Xu et al., 2020).

When the SARS-COV-2 virus enters the body, it can rapidly activated CD4^+^T lymphocytes to become T helper 1 (Th1) cells to secrete pro-inflammatory factors (e.g., granulocyte-macrophage colony-stimulating factor (GC-SF), IFN-γ, IL-2, IL-7, IL-6.). While the GC-SF further activates CD14^+^CD16^+^ inflammatory monocytes, producing a large number of IL-6, TNF-α and other cytokines in Figure 1 (Yonggang et al., 2020). The release of pro-inflammatory factors i s consistent with the activation of NLRP3/inflammasomes (Chen et al., 2019). During COVID-19 infection, SARS-CoV proteins E and 3a IC induces Ca2^+^ efflux and activates NLRP3/inflammasome complex, which in turn, triggers zymogen procaspase-1 to convert into the active caspase-1 and eventually establish the inflammatory caspase (Farag et al., 2020). Inactive pro-IL-1βand pro-IL-18 were converted to active pro-inflammatory cytokines (such as IL-1βand IL-18) (Franchi et al., 2012). Franchi et al. (2014) found that when pathogen-associated molecular patterns (PAMPs) or danger-associated molecular patterns (DAMPs) were recognized by Toll-like receptors (TLRs), activating the nuclear factor kappa B (NF-κB) -mediated signaling pathways, and thus up-regulated the transcription of inflammasomal-related components (Epstein and Dinarello, 1984). Ultimately, it stimulates the release of TNF and IL-6 and increases levels of TNF-α, IL-13, IL-4, and IL-8, contributing to acute pro-inflammatory response in Figure 1 (Martinon and Tschopp, 2007; Davis et al., 2011; Luigi and Gabriel, 2012; Hirano and Murakami, 2020). Hirano and Murakami (2020) proposed that the AngII pathway caused potential mechanisms for cytokine storms When the SARS-COV-2 virus engulfed ACE2, the content of ACE2 on cells decreased and stimulated the ACE/Ang II/AT1R axis through pattern-recognition receptors to activate NF-κB and disintegrin and metalloprotease 17 (ADAM17). ADAM17 can generate TNF-α, induction IL-6Rα to the form soluble form (sIL-6Rα), and then mediated by gp130-mediated to forms sIL-6Rα-IL-6 complexes, ultimately activate signal transducer and activator of transcription 3 (STAT3). Both NF-κB and STAT3 can activate IL 6 amplifier (IL-6 Amp), which in turn causes various pro-inflammatory cytokines and chemokines (Figure 1).

Cigarette smoke is known to cause abnormal inflammatory activation of the bronchial epithelium and promote damage to macrophage function, increasing the risk of infection (Hiemstra, 2013; Aghapour et al., 2018). More and more studies suggest cigarette smoke activates the NLRP3 inflammasome in the lung epithelium, increasing the expression of NLRP3, pro-IL-1β, and caspase-1, enhancing the activity of Caspase-1 and the release of inflammasome related cytokines IL 1β and IL 18 in Figure 1 (Li et al., 2016; Yi et al., 2018). Buscetta et al. (2020) found that cigarette smoke extract could increase the activity of caspase-1 through an NLRP3-independent and TLR4-TRIF-caspase-8-dependent pathway, leading to a decrease in basic glycolytic flux and response to lipopolysaccharide, which may eventually lead to macrophage dysfunction and increase the risk of infection in smokers. Simultaneously, in the context of subchronic electronic cigarette exposure, the body increases the release of pro-inflammatory factors and ACE2 receptors by regulating nAChRα7, leading to an inflammatory response and dysregulated repair (Wang Q. et al., 2020). The De Cunto team (De Cunto et al., 2016) showed that, under chronic smoking conditions, the expression of some pro-inflammatory cytokines was upregulated (e.g., Il-6, TNF-α, KC).

Paradoxically, nicotine was indicated to be an agonist of the cholinergic anti-inflammatory pathway. It has anti-inflammatory properties and can regulate the body’s immune response. It can also inhibit the release of pro-inflammatory cytokines (e.g., TNF, IL-1, IL-6) without inhibiting the release of anti-inflammatory cytokines (e.g., IL-10) (Wang H. et al., 2003). Because of long-term chronic respiratory infection, the level of antibodies against streptococcus pneumoniae in smokers’ bodies will increase, making it possible to have certain anti-inflammatory capabilities (Sankilampi et al., 1997; Nuorti et al., 2000). The most effective evidence supporting the therapeutic anti-inflammatory potential of nicotine is the epidemiological study of smokers with ulcerative colitis; 90% of patients with ulcerative colitis are non-smokers, and the symptoms of colitis in smokers who smoke intermittently can be improved when smoking (Jenkins et al., 2005). Nicotine has been used in clinical trials to treat ulcerative colitis; however, its non-specific effects and collateral toxicity limit its clinical use (Ghia et al., 2006; de Jonge and Ulloa, 2007). At present, there is no certain evidence that nicotine increases anti-COVID-19 antibody levels in humans, and the mechanism of its action remains unclear.

These contradictions may be related to the concentration of nicotine. Stable nicotine dose therapy has good anti-inflammatory effects. However, smoking can cause a continuous increase in plasma nicotine concentration, resulting in addiction and adverse consequences. The WHO also stated that there is not enough information to confirm any connection between tobacco or nicotine in the prevention or treatment of COVID-19. Therefore, smoking is by no means a protective measure against COVID-19. Doctors and public health professionals should collect accurate smoking data, and further independent studies are necessary to analyze the impact of smoking on the incidence rate, progression, and COVID-19 mortality.

## Tobacco Use and COVID-19 Virus Transmission

The COVID-19 virus can infect individuals of any age. The virus has a wide range of transmission methods. It may spread through contact, droplets, air, pollutants, faces, etc.; however, its main mode of transmission is respiratory droplets. In addition to contact transmission, the source can be respiratory secretions, droplets, aerosols and contaminated surfaces (Hui et al., 2020). People with or without symptoms can transmit the virus (Nair et al., 2017). Therefore, strict cross-infection control measures are difficult to maintain due to smoking behavior (Wang D. et al., 2020). Smokers’ hands and cigarette filters are contaminated by SARS-CoV-2 in many ways. Smokers gather together in closed environments where they chat, drink, and pass cigarettes to other smokers, which increases the risk of COVID-19 infection (Figure 2). In the meantime, cigarette smoke causes users to cough or sneeze. This process produces large amounts of aerosols containing SARS-CoV-2, which can remain in the air for 3 h in the form of droplets or micro-aerosols, and they can survive as aerosols and on the surface (plastic paper and steel) for several hours to several days (Sherman, 1991; Benjamin, 2011). Even if strict social distance policies are maintained, contaminated humans may still be infected. Also, smokeless tobacco products may contribute to the COVID-19 epidemic and increase susceptibility (Singh and Chaturvedi, 2020). Users put smokeless tobacco products in their mouths, chew repeatedly, and finally spit them out along with saliva, which contains a variety of pathogens, including SARS-CoV-2. Smokeless tobacco users may exhibit behavior like spitting, and they often exhibit respiratory symptoms, cancer, and other diseases at high incidence rates, which are closely related to the COVID-19 epidemic (Siddiqi et al., 2015). Therefore, smokers’ social culture may promote the spread of the virus.

## COVID-19 Pandemic and Smoking Cessation

Do the home isolation policies implemented during the COVID-19 pandemic increase smokers’ motivation to quit? Heerfordt and Heerfordt (2020) investigated the global search trend of smoking cessation information during the COVID-19 pandemic (January–April 2020) from the perspective of Google’s hot search trend and found that the search volume of smoking cessation information on Google did not increase significantly (Tieks et al., 2019). In contrast, because of the prolonged home isolation and regional blockade during the pandemic, residents experienced a series of symptoms, including panic, anxiety, sleep disturbance, etc. (Banerjee, 2020). This isolation may also have had a certain impact on nicotine addicts (Pfefferbaum and North, 2020), driving them to use substances, especially tobacco or alcohol, to relieve stress and negative emotions (García-Álvarez et al., 2020). One report showed that smokers’ daily smoking rates increased by 49.9% during the epidemic, which could explain why the tobacco industry was unaffected during such a severe epidemic (Tobacco Reporter, 2020). A survey of smoking behavior and psychological dynamics in Italy in April 2020 (including 1,825 participants) showed that staying at home caused most exclusive smokers to consider quitting; however, most e-cigarette users did not consider stopping e-cigarettes use, and cigarettes and e-liquid purchases increased; one-third of former smokers considered relapse (Caponnetto et al., 2020). Although the governments around the world was not very active regarding efforts to encourage smoking cessation during the epidemic, the government can still encourage smokers to quit and maintain their health.

To date, many governments and health institutions have not included smokers in the high-risk population for COVID-19 and seldom issue warnings regarding the effects of smoking on COVID-19’s spread. With the studies on the relationship between smoking and COVID-19, this situation may change.

## Summary

This review clearly indicate the complexity of the relationship between smoking and COVID-19. Smoking can increased mucosal permeability, reducing airway purification function and the screening of harmful microorganisms, increasing the risk of viral of respiratory infections and aggravating the severity of respiratory diseases among smokers. There is also growing evidence to support the WHO’s conclusion that smokers are at a higher risk of severe COVID-19 symptoms. Meanwhile, ongoing smoking can aggravate the condition of COVID-19 patients and increase the risk of death. Smokers’ social behaviors, such as gathering to smoke, can increase of COVID-19 transmission. Although the mechanism of nicotine’s effect on ACE2 expression needs further study, upregulation of ACE2 expression can increase the susceptibility to and risk of COVID-19. Also, nicotine can increase pneumococcal and streptococcal antibody levels to an extent. However, there is insufficient evidence to indicate that there is any link between nicotine in tobacco and the prevention or treatment of COVID-19. Smoking is not a measure to prevent COVID-19. Therefore, governments and health care providers should identify the importance of tobacco control, especially during the COVID-19 epidemic. Public health messages should increase publicity about tobacco hazards, the benefits of quitting, and provide strict, safe, and healthy tobacco management in public and the workplace to encourage smokers to quit (Figure 2).

## Author Contributions

JX and RZ wrote this review. WW and YZ supervised the entire work and critically revised the manuscript. OC read and amended the final manuscript. All authors contributed to the article and approved the submitted version.

## Conflict of Interest

The authors declare that the research was conducted in the absence of any commercial or financial relationships that could be construed as a potential conflict of interest.
